# Mid-thigh circumference as an indicator of nutritional status to predict adverse pregnancy outcomes among HIV-infected and HIV-uninfected women in Malawi

**DOI:** 10.1186/s12884-021-04118-4

**Published:** 2021-09-23

**Authors:** Keerthana Hirudayakanth, Luis Gadama, Sufia Dadabhai, Chaplain Katumbi, Hazzie Mvula, Bonus Makanani, Frank Taulo, Taha E. Taha

**Affiliations:** 1 Johns Hopkins Bloomberg School of Public Health, 615 N Wolfe Street, Baltimore, MD 21205 USA; 2 College of Medicine, University of Malawi, Blantyre, Malawi; 3 College of Medicine-Johns Hopkins Research Project, Blantyre, Malawi

**Keywords:** Mid-thigh circumference, Body-mass index, Maternal anthropometry, Pregnancy outcomes, HIV infection, Malawi

## Abstract

**Background:**

High rates of adverse pregnancy outcomes globally raise the need to understand risk factors and develop preventative interventions. The Pregnancy Outcomes in the Era of Universal Antiretroviral Treatment in Sub-Saharan Africa (POISE Study) was a prospective, observational cohort study conducted from 2016 to 2017 in Blantyre, Malawi. We examine the associations between indicators of nutritional status, specifically mid-thigh circumference (MTC) and body-mass index (BMI), and adverse pregnancy outcomes, low birth weight (LBW), preterm birth (PTB), and small-for-gestational age (SGA), in a cohort of HIV-infected and HIV-uninfected women.

**Methods:**

Sociodemographic, clinical, laboratory, and maternal height, weight and MTC data were collected immediately before or after delivery at the Queen Elizabeth Central Hospital (QEHC) and 4 affiliated health centers in Blantyre, Malawi. LBW was defined as birth weight < 2.5 kg; PTB as gestational age < 37 weeks using Ballard score; and SGA as birth weight < 10th percentile for gestational age. Descriptive, stratified, and multivariable logistic regression were conducted using R.

**Results:**

Data from 1298 women were analyzed: 614 HIV-infected and 684 HIV-uninfected. MTC was inversely associated with LBW (adjusted odds ratio [aOR] = 0.95, *p* = 0.03) and PTB (aOR 0.92, *p* < 0.001), after controlling for HIV status, age, socioeconomic status and hemoglobin. The association between MTC and SGA was (aOR 0.99, *p* = 0.53). Similarly, higher BMI was significantly associated with lower odds of PTB (aOR 0.90, *p* < 0.001), LBW (aOR 0.93, *p* = 0.05), and SGA (aOR 0.95, *p* = 0.04).

**Conclusions:**

We observed an inverse relationship between MTC and adverse pregnancy outcomes in Malawi irrespective of HIV infection. MTC performs comparably to BMI; the ease of measuring MTC could make it a practical tool in resource-constrained settings for identification of women at risk of adverse pregnancy outcomes.

## Background

Maternal nutrition impacts both the health of the mother and the child, raising the importance of assessing the nutritional status of pregnant women as part of prenatal care. Existing evidence affirms a strong link between maternal nutritional status and adverse pregnancy outcomes such as low birth weight (LBW), preterm birth (PTB) and small for gestational age (SGA) infants in both HIV-infected and HIV-uninfected women in sub-Saharan Africa [1–3]. Specifically, among HIV-infected women, HIV-induced wasting or weight loss is common during disease progression. Research among HIV-infected women in Tanzania, Zambia, and Malawi suggested links between poor maternal nutrition and increased adverse pregnancy outcomes [1]. Weight loss from any cause, including inadequate intake, impacts the nutritional status of women, raising the need for effective and accessible tools to screen women in low-resource settings to avoid adverse pregnancy outcomes.

Previous studies have used body-mass-index (BMI) or weight gained during pregnancy as an anthropometric measure of nutritional status to predict adverse pregnancy outcomes, however, there are concerns around the effectiveness of this measurement. Anthropometric measures such as maternal height and weight, weight gain during pregnancy, and body mass had low specificity and sensitivity to detect low birth weight and also had low diagnostic value for predicting SGA infants [4, 5]. BMI does not consider body composition and or muscle wasting, which is an important consideration for HIV-infected women, as muscle wasting is a common effect during the progression of the disease. Research support the use of other anthropometric measures such as muscle area measurements to predict pregnancy outcomes, as weight and BMI may not be the most suitable indicator of muscle and fat stored in the body. An observational study conducted among HIV-infected women in Tanzania demonstrated the usefulness of assessing maternal nutritional status using measurements such as mid-arm muscle area (MAMA) and mid-upper arm circumference (MUAC) to screen for adverse pregnancy outcomes of LBW, PTB, and SGA. Findings showed an inverse relationship between higher values of MAMA and MUAC and lower risk of these adverse pregnancy outcomes [6]. Among HIV-infected women in Malawi, mid-upper arm circumference (MUAC), arm muscle area (AMA), and arm fat area (AFA) were all shown to be negatively associated with LBW [7]. While MUAC and MAMA have been used as indicators of nutritional status, mid-thigh circumference (MTC) measurement has not been frequently assessed to determine associations between nutritional status and birth outcomes among HIV-infected and uninfected women.

Mid-thigh circumference (MTC) as an indicator of nutritional status has previously been shown to be a reliable predictor of mortality and could be a valuable tool to predict adverse pregnancy outcomes [8, 9]. There are key advantages of using MTC compared to BMI as a maternal anthropometric measurement of nutritional when predicting adverse birth outcomes. Research among HIV-infected patients who have muscle wasting and central adiposity supports the use of MTC over BMI to assess the risk of mortality [10]. MTC was shown to be stronger predictor of loss of subcutaneous fat or lipoatrophy compared to other anthropometric tools among HIV-infected women in South Africa on antiretroviral treatment (ART) [11]. Lower body muscle wasting has also been characteristic of chronic obstructive pulmonary disease (COPD), and measurement of the mid-thigh muscle cross-sectional area was found to be a better predictor of mortality than BMI among patients with COPD [9]. Additionally, the ease of measurement and lower burden of resources are also advantages of using MTC in low-resource clinical settings, where calibrated scales and equipment to measure weight and height as well as the calculation of an index for BMI are not required. Therefore, using MTC may potentially be better than BMI as an anthropometric screening indicator as it may be a more suitable measure of muscle and fat composition, and is easier to obtain.

The “Pregnancy Outcomes in the Era of Universal Antiretroviral Treatment in Sub-Saharan Africa (POISE Study)” was conducted in Blantyre, Malawi during 2016–2017 to determine the pregnancy outcomes of LBW, PTB and SGA among HIV-infected women on life-long ART and among a comparable cohort of HIV-uninfected women [12]. The current secondary analysis is based on data from the POISE study and aims to assess the association of mid-thigh circumference (MTC), as a nutritional indicator, with the pregnancy outcomes of LBW, PTB and SGA. While BMI has been more frequently used in prior studies, given its limitations, the objective of this study is to investigate whether MTC is a reasonable anthropometric measure of nutritional status to predict adverse pregnancy outcomes.

## Methods

The parent POISE study was conducted at the Queen Elizabeth Central Hospital (QEHC) and 4 affiliated health centers in Blantyre, Malawi from January 2016 to September 2017. At these health facilities, eligible women were screened and enrolled in the study at delivery. Full details of the study have been published [12]. Briefly, POISE was a prospective observational study. Women were enrolled at the five health facilities at time of delivery (before or after delivery) and followed for one year post-delivery with their infants. Inclusion criteria were the following: confirmed HIV status, written informed consent and live singleton births. Participants were excluded if they were unable to provide informed consent or had multiple births. HIV-infected women were eligible for the study if they had a CD4 cell count ≥ 350 cells/mm^3^, were on ART for at least 1 week before delivery and did not have WHO stage 3 or 4 HIV disease. The parent study aimed to assess the impact of ART among clinically healthy HIV-infected women (i.e., they did have low CD4 cell count or stage 3 or 4 HIV disease stage). Participants were counseled and consented to enroll along with their infants. Eligible HIV-uninfected women were concurrently enrolled in the same health facilities at which the HIV-infected women were enrolled.

Trained study nurses administered structured questionnaires and conducted physical examinations after obtaining informed consent at delivery. The questionnaires collected sociodemographic characteristics, medical history, and sexual and reproductive health information, which included information on risk factors and potential confounders. For HIV-infected women, additional information on ART use and adherence was included and blood samples were collected to measure the HIV viral load and CD4 cell count. The physical examination of all women following delivery included anthropometric measurements of height, weight, and MTC, which were the primary anthropometric indicators in this study. Weight was measured to the nearest 0.1 kg; height and MTC were measured to the nearest 0.5 cm. Body mass index (BMI) was calculated as kg/m^2^ using the height and weight measurements. Measurement of MTC was conducted by trained study workers on the right thigh while the woman was standing using a flexible measuring tape. The mid-point between the top of the femur and the knee was marked and MTC was determined in centimeters. Based on previous research conducted in Malawi, women who reported having electricity in the home were identified as having high socioeconomic status and women without electricity in the home were identified as having low socioeconomic status [12]. After birth, a physical examination of the infant was conducted, which included birth weight and other anthropometric measurements. Gestational age was calculated using the Ballard score within 36 h of birth by a trained study nurse. Gestational age was estimated using the date of last menstrual period when the Ballard score was missing. Estimates based on Ballard score and date of last menstrual period were compared to assess potential misclassifications [13].

Data were entered using a database deisgned in Microsoft Access and data cleaning was done in a regular interval in Microsoft Excel and Stata (version 14.2). Data double entry was done and anonymized prior to analysis.

The primary outcomes of this study were PTB, LBW, and SGA. PTB was defined as a gestational age < 37 completed weeks. LBW was defined as birth weight < 2.5 kg. SGA was defined as birth weight less than the 10^th^ percentile for gestational age, using the reference population as described by Oken et. al [14]. Descriptive analyses were conducted first on the sociodemographic and clinical data and primary outcomes of interest. Stratified analyses, graphical presentations, and data transformations were also used. We first assessed the associations between MTC and the outcomes of birth weight and gestational age as continuous variables using linear regression models. We modeled the associations between MTC and the three pregnancy outcomes (PTB, LBW, and SGA as binary outcomes) using univariable and multivariable logistic regression analyses, which controlled for potential cofounders. Univariable and multivariable models were also created to examine the associations between BMI and the three pregnancy outcomes. The following baseline risk factors were controlled for in the multivariable analyses: HIV status (infected/uninfected; all HIV-infected women were taking the standard national ART regimen in Malawi at the time of study conduct [tenofovir, lamivudine and efavirenz]), maternal age in years (continuous), hemoglobin level (< 10 g/dL/ ≥ 10 g/dL) and socioeconomic status measured as having electricity at home (yes/no). These were selected based on the risk factors assessed in the parent study, which were of biological and epidemiological importance [12]. The final multivariable model included these potential confounders in addition to the exposure of interest– MTC. The analyses were run at a *p* < 0.05 significance level, and crude and adjusted odds ratios with 95% confidence intervals are presented. All analyses were done in R statistical software package version 3.6.3 (Vienna, Austria).

## Results

A total of 1299 women met the inclusion criteria, provided consent, and enrolled in the parent (original) POISE study [12]. One participant was excluded in this secondary analysis because the MTC measurement recorded in the dataset was an outlier. Therefore, this analysis includes 1298 participants: 614 HIV-infected women on lifelong ART and 684 HIV-uninfected women. The baseline characteristics of the participants included in this analysis are shown in Table 1. The median age of HIV-infected women was 28.7 years and of HIV-uninfected women was 24.9 years (*p* < 0.001). The number of pregnancies was higher in the HIV-infected women compared to the HIV-uninfected women (3.4 vs. 2.5 pregnancies, *p* < 0.001). The mean MTC for the HIV-infected women was 48.3 cm and for the HIV-uninfected women 48.3 cm (*p* = 0.66). The mean maternal BMI of HIV-infected women was 23.7 kg/m^2^ and for HIV-uninfected women 24.1 kg/m^2^ (*p* = 0.04). Among the 1298 participants, 6.0% had LBW infants, 10.0% had PTB infants, 17.8% had SGA infants. The differences in adverse pregnancy outcomes between HIV-infected and uninfected were not statistically significant (Table 1).

**Table 1:** Baseline Characteristics of study population, stratified by HIV status

Characteristics	HIV- infected (*n* = 614)	HIV-uninfected (*n*-684)	Total (*n* = 1298)	*P*-value^a^
*Mean (standard deviation)*				
Maternal Age (years)	28.69 (5.98)	24.87 (5.44)	26.67 (6.01)	< 0.001
Maternal Body Mass Index (BMI) (kg/m^2^)	23.73 (3.45)	24.14 (3.70)	23.95 (3.59)	0.04
Maternal Mid-thigh circumference (MTC) (cm)	48.26 (5.60)	48.40 (5.89)	48.33 (5.75)	0.66
Gravidity	3.37 (1.57)	2.51 (1.54)	2.92 (1.61)	< 0.001
Birth weight (kg)	3.04 (0.41)	3.06 (0.38)	3.05 (0.39)	0.41
*count (%)*				
Hemoglobin level < 10 g/dL	156 (25.4)	70 (10.3)	226 (17.40)	< 0.001
No electricity in the house^b^	321 (52.3)	340 (49.7)	661 (50.90)	0.38
Preterm (< 37 weeks) (best available measure)	65 (10.6)	65 (9.5)	130 (10)	0.58
Small for gestational age (< 10^th^ percentile)	105 (17.1)	126 (18.4)	231 (17.8)	0.58
Low birth weight (< 2·5 kg) count (%)	44 (7.2)	35 (5)	78 (6)	0.12

^a^Two sample t-test for continuous variables; χ^2^ test for categorical variables

^b^ Indicator of socioeconomic status

**Fig. 1:**
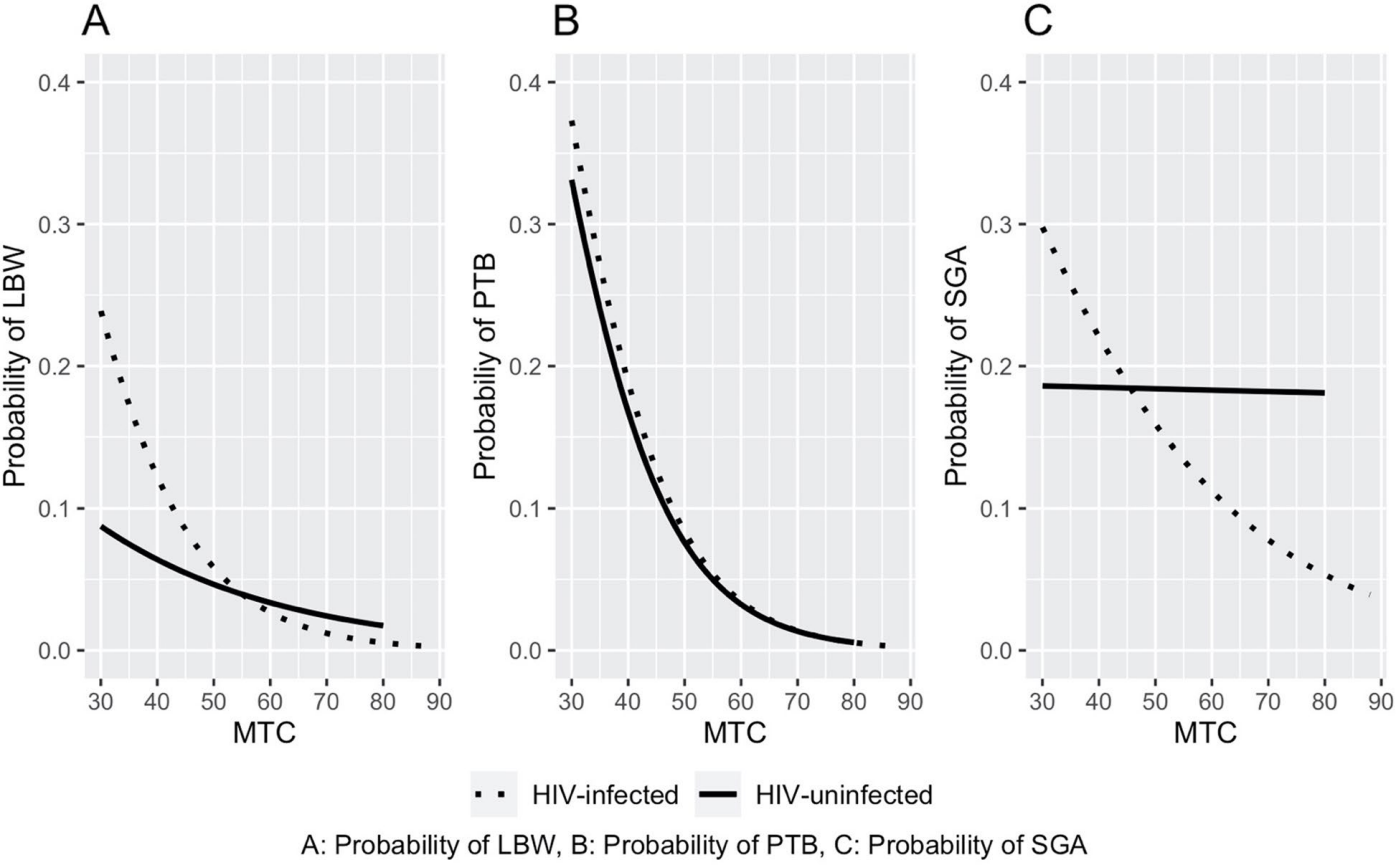
Probability Plots Of Logistic Regression For LBW, PTB, and SGA for HIV-Infected and HIV-Uninfected Women

There was a linear relationship between MTC and birth weight outcome (regression coefficient = 0.01; *p* < 0.001). There was a similar linear relationship between MTC and gestational age (regression coefficient = 0.04; *p* < 0.001). The same linear relationship was also observed between BMI and birth weight (regression coefficient = 0.02; *p* < 0.001), and BMI and gestational age (regression coefficient = 0.04; *p* < 0.001). Figure 1 shows the probabilities obtained from a logistic regression model for LBW, PTB and SGA with changes in MTC in centimeters as a continuous measurement. All three figures show a decreasing trend in the probability of adverse pregnancy outcomes as MTC increases. In Fig. 1A, the probability of LBW among HIV-infected women is higher than that of HIV-uninfected women at lower MTC values, however, the plots approach the same probability around MTC of 54–55 cm. In Fig. 1B, the probability plots of PTB among HIV-uninfected and -infected women appears to be similar. Figure 1C shows higher probabilities of SGA among infants of HIV-infected women at lower MTC values, and the plots approach the same probability around MTC of 45–46 cm. The probability of SGA among HIV-uninfected women is roughly constant (with a slight downward trend). The probability of SGA among HIV-infected women follows the pattern with LBW and PTB; with increase in MTC, there is a decrease in the probability of SGA.

Table 2 shows the association between adverse pregnancy outcomes, our exposure variable of interest MTC, as well as other variables included in the univariable and multivariable analyses. In the univariable analyses, there were statistically significant associations between MTC and LBW (*p* = 0.01) and PTB (*p* < 0.001). There were significant associations between electricity in the home (an indicator of socioeconomic status) and LBW (*p* = 0.009) and PTB (*p* < 0.001). In the univariable analysis, there was also a statistically significant association between age and SGA (*p* = 0.001). The multivariable results showed significant associations between MTC and the outcomes of LBW (aOR = 0.95, *p* = 0.03, 95% CI 0.91–0.99) and PTB (aOR = 0.92, *p* < 0.001, 95% CI 0.88–0.95). In the multivariable model for SGA, age was statistically significant; *p* = 0.001. The association between socioeconomic status and the three adverse pregnancy outcomes were statistically significant in the multivariable models. The same univariable and multivariable analyses used in Table 2 were also conducted with the variable of interest MTC dichotomized to low (less than or equal to median MTC) or high (greater than median MTC). These results were similar to those in Table 2.

**Table 2:** Association of mid-thigh circumference (MTC) with adverse pregnancy outcomes

Risk Factors	Model 1: LBW						Model 2: PTB						Model 3: SGA					
	**OR**^**a**^	***P*-value**	**95% CI**^**b**^	**aOR**^**a**^	***P*-value**	**95% CI**^**c**^	**OR**^**a**^	***P*-value**	**95% CI**^**b**^	**aOR**^**a**^	***P*-value**	**95% CI**^**c**^	**OR**^**a**^	***P*-value**	**95% CI**^**b**^	**aOR**^**a**^	***P*-value**	**95% CI**^**c**^
MTC (cm)	0.94	0.01	0.90–0.99	0.95	0.03	0.91–0.99	0.91	0.001	0.88–0.95	0.92	0.001	0.88–0.95	0.98	0.19	0.96–1.01	0.99	0.53	0.97–1.02
HIV (ref cat: HIV-uninfected)	1.48	0.1	0.93–2.35	1.66	0.046	1.01–2.72	1.13	0.52	0.78–1.62	1.15	0.48	0.78–1.71	0.91	0.54	0.69–1.21	1.05	0.78	0.77–1.43
Age (yrs)	0.98	0.39	0.94–1.02	0.97	0.16	0.93–1.01	1.00	0.86	0.97–1.03	1.00	0.84	0.97–1.03	0.96	0.001	0.94–0.98	0.96	0.001	0.93–0.98
Hemoglobin level (ref cat: ≥ 10 g/dL)	1.04	0.90	0.55–1.83	0.87	0.67	0.47–1.61	0.96	0.87	0.58–1.53	0.83	0.46	0.50–1.37	1.10	0.60	0.76–1.58	1.11	0.60	0.76–1.61
Electricity at home^d^ (ref cat: Yes)	1.89	0.009	1.18–3.09	1.79	0.02	1.10–2.91	2.09	0.001	1.44–3.09	1.90	0.001	1.29–2.80	1.33	0.053	1.00–1.77	1.35	0.04	1.01–1.80

Adjusted risk factors: HIV infection status, age, hemoglobin level, and electricity at home (socioeconomic status)

^a^*OR*  Crude odds ratio, *aOR* Adjusted odds ratio

^b^ 95% Confidence Interval for the OR

^c^ 95% Confidence Interval for the aOR

^d^ Indicator of socioeconomic status

Table 3 shows the association between adverse pregnancy outcomes, BMI, as well as other variables included in the univariable and multivariable analyses. In the univariable analyses there were statistically significant associations between BMI and LBW (*p* = 0.01), PTB (*p* < 0.001), and SGA (*p* = 0.006). Electricity in the home (socioeconomic status) was associated with LBW (*p* = 0.009) and PTB (*p* < 0.001). Age had a statistically significant association with SGA (*p* = 0.001). After controlling for risk factors, the multivariable analyses showed statistically significant associations between BMI and PTB (*p* < 0.001) and SGA (*p* = 0.04). There were also statistically significant associations between electricity and LBW (*p* = 0.02) and PTB (*p* < 0.001) in the multivariable models. Age was associated with SGA in the multivariable analyses (*p* = 0.002). We also did these same analyses as in Table 3 with BMI dichotomized to low (less than or equal to median BMI) or high (greater than median BMI). These results were similar to those in Table 3.

**Table 3:** Association of body-mass index (BMI) with adverse pregnancy outcomes

Risk Factors	Model 1: LBW						Model 2: PTB						Model 3: SGA					
	**OR**^**a**^	***P*-value**	**95% CI**^**b**^	**aOR**^**a**^	***P*-value**	**95% CI**^**c**^	**OR**^**a**^	***P*-value**	**95% CI**^**b**^	**aOR**^**a**^	***P*-value**	**95% CI**^**c**^	**OR**^**a**^	***P*-value**	**95% CI**^**b**^	**aOR**^**a**^	***P*-value**	**95% CI**^**c**^
BMI (kg / m2)	0.91	0.01	0.84–0.98	0.93	0.050	0.86–1.00	0.89	0.001	0.84–0.94	0.90	0.001	0.84–0.96	0.94	0.006	0.90–0.98	0.95	0.04	0.91–1.00
HIV (ref cat: HIV-uninfected)	1.48	0.1	0.93–2.35	1.61	0.06	0.98–2.65	1.13	0.52	0.78–1.62	1.10	0.65	0.74–1.63	0.91	0.54	0.69–1.21	1.02	0.90	0.74–1.39
Age (yrs)	0.98	0.39	0.94–1.02	0.97	0.18	0.93–1.01	1.00	0.86	0.97–1.03	1.00	0.88	0.97–1.03	0.96	0.001	0.94–0.98	0.96	0.002	0.94–0.99
Hemoglobin level (ref cat: ≥ 10 g/dL)	1.04	0.90	0.55–1.83	0.89	0.71	0.48–1.64	0.96	0.87	0.58–1.53	0.87	0.58	0.53–1.42	1.10	0.60	0.76–1.58	1.09	0.64	0.75–1.60
Electricity at home^d^ (ref cat: Yes)	1.89	0.009	1.18–3.09	1.81	0.02	1.12–2.94	2.09	0.001	1.44–3.09	1.96	0.001	1.33–2.89	1.33	0.053	1.00–1.77	1.31	0.07	0.98–1.76

Adjusted risk factors: HIV infection status, age, hemoglobin level, and electricity at home (socioeconomic status)

^a^*OR*  Crude odds ratio, *aOR* Adjusted odds ratio

^b^ 95% Confidence Interval for the OR

^c^ 95% Confidence Interval for the aOR

^d^ Indicator of socioeconomic status

## Discussion

In this study, we assessed the association between MTC, as an anthropometric measurement of nutritional status, and pregnancy outcomes among HIV-infected and HIV-uninfected women in Blantyre (urban), Malawi. There was a linear relationship between MTC and birth weight, and MTC and gestational age. There was a similar trend in the relationship between MTC and all three adverse pregnancy outcomes: an increase in MTC was associated with a decrease in LBW, PTB, and SGA. These associations were statistically significant between MTC and LBW and PTB after adjusting for HIV infection and other factors. However, the association between MTC and SGA was not statistically significant in multivariable analyses; examination of univariable data showed the trends of SGA decline with increase in MTC was more apparent in HIV-infected than in HIV-uninfected women. There was also a lack of association between HIV infection and LBW, SGA, and PTB in multivariable analyses after adjusting for MTC and other factors. The findings show that socioeconomic status was a consistent risk factor associated with adverse pregnancy outcomes after adjustment, highlighting the complex role other unmeasured factors (e.g., income, availability of food, seasonality, other infections) can play in these resource-constrained settings.

Most previous studies used BMI as an indicator of nutritional status and to assess chronic energy deficiency (CED) and undernutrition in multiple settings.[15, 16] In the current analysis, both BMI and MTC were associated with adverse pregnancy outcomes in the same population; higher BMI and MTC were associated with lower adverse pregnancy outcomes. Similar to the association between MTC and adverse outcomes in Table 2, there were statistically significant inverse associations between BMI and adverse pregnancy outcomes after controlling for other risk factors in Table 3. Likewise, we note the lack of association with HIV infection and the consistent association of these adverse outcomes with socioeconomic status.

Since MTC performed similarly to BMI as a maternal anthropometric measure of nutritional status, it is important to consider the added benefits of using MTC as a tool in low-income settings. The ease of measuring MTC and appropriateness for use in a primary care clinic or busy outpatient centers of tertiary hospitals makes it a practical screening option in resource-constrained settings, where scales, particularly calibrated scales, are not consistently available. Compared to BMI, which requires measures of height, weight, and calculation of an index, MTC may be more accurate and simpler. Additionally, BMI during pregnancy (or immediate postpartum) may be inaccurate because weight changes during pregnancy, especially during the third trimester when many African women attend their first antenatal care visit. Swelling due to fluid retention involves mostly the feet and hands; therefore, assessment of MTC may be a reasonable measure.

Other studies point out the limitations of BMI as an anthropometric measurement as it does not consider body composition. An important advantage of using MTC is that it can be used to measure muscle mass, which is not fully captured by BMI [8–10]. A prospective cohort study among Danish men and women found that lower thigh circumference was associated with an increased risk of cardiovascular disease and mortality, which may be associated with low muscle mass in the area [8]. Research among patients with chronic obstructive pulmonary disease found that midthigh muscle cross-sectional area was a better predictor of mortality and supported its use over the current reliance on BMI in clinical settings because it takes body composition and muscle wasting into account [9]. This is especially important among HIV-infected patients, since muscle wasting is a common occurrence during the progression of HIV disease. One study showed that lower muscle mass and higher central adiposity were linked to higher mortality among HIV-infected patients [10]. This study used different anthropometric measurements and supported the use of MTC over BMI to assess the risk of mortality among HIV-infected patients who have both muscle wasting and increased central adiposity. In this study, BMI was not as sensitive to changes in body composition (particularly due to wasting) and underestimated the risk of mortality. This underscores the importance of considering alternative anthropometric measurements such as MTC to evaluate the risk of adverse outcomes [10]. Previous studies in Malawi and Tanzania confirmed the association between nutritional status using the anthropometric measurements of mid-upper arm circumference and mid-arm muscle area and adverse pregnancy outcomes [6, 7]. Another study conducted in South Africa also showed that the median thigh circumferences among HIV-infected women in different ART treatment groups were higher than measurements observed in this study (56.0 cm in ART naïve and 57.0–59.0 cm in ART users) [17]. The median MTC values among the participants of our study were 48.00 cm in HIV-infected and HIV-uninfected women.

The lack of association of adverse pregnancy outcomes with HIV infection is likely the result of the beneficial maternal health effects due to ART use by HIV-infected women (LBW was nonetheless higher in HIV-infected women compared to HIV-uninfected – borderline statistical significance as shown in Table 2) [12]. While the lack of association between HIV infection and LBW, PTB and SGA could be due to beneficial effects of ART, another explanation could be related to recruitment and selection issues. This includes the exclusion of women and neonates with severe complications and/or adverse outcomes, as well as HIV-infected women with low CD4 count and severe clinical disease– resulting in fewer differences between HIV-infected and HIV-uninfected women, which may have led to bias towards the null. The study aimed to assess the pregnancy outcomes among HIV-infected women who were healthy to understand the effect of ART in minimizing the impact of adverse outcomes. The hypothesis of the parent study was that ART reduces differences in pregnancy outcomes between the HIV-infected and HIV-uninfected cohorts [12].

Considering the potential for selection bias, the same recruitment study procedures were conducted for both the HIV-infected and HIV-uninfected women, therefore selection would likely impact both groups similarly and result in minimal bias. In this study, both Ballard Score and LMP was used to determine rates pregnancy outcomes and compared to reduce the possibility of misclassification. It was found that LMP overestimated PTB, however, there were no observable differences between the HIV-infected and HIV-uninfected groups. We also acknowledge measurement of gestational age using Ballard Score or LMP is difficult in these settings. Residual confounding due to factors not included in the multivariable models may have also impacted the findings, such as history of previous preterm births and conditions such as diabetes and hypertensive disease.

While our findings further strengthen the association between MTC and adverse pregnancy outcomes and its potential usefulness as a tool for assessing nutritional status, this study did not evaluate the sensitivity and specificity of MTC. A limitation of the analysis is our inability to determine a specific cutoff value of MTC for LBW, PTB, SGA outcomes, which may have been due to less variability in the study population based on the exclusion criteria of the parent study. Nonetheless, use of MTC as a screening anthropometric indicator of nutritional status appears appropriate and could overcome some limitations of commonly used measures during pregnancy. Our main study showed that the rates of adverse pregnancy outcomes remain high among HIV-infected and HIV-uninfected women in Malawi [12]. Therefore, assessment using simple tools such as MTC measurement and appropriate interventions should be a priority irrespective of HIV infection in antenatal clinics in these settings.

## Data Availability

The datasets used and/or analysed during the current study are available from the corresponding author on reasonable request.
